# The Design and Preclinical Evaluation of a Single-Label Bimodal Nanobody Tracer for Image-Guided Surgery

**DOI:** 10.3390/biom11030360

**Published:** 2021-02-26

**Authors:** Pieterjan Debie, Noemi B. Declerck, Danny van Willigen, Celine M. Huygen, Bieke De Sloovere, Lukasz Mateusiak, Jessica Bridoux, Janik Puttemans, Nick Devoogdt, Fijs W. B. van Leeuwen, Sophie Hernot

**Affiliations:** 1 Laboratory for In Vivo Cellular and Molecular Imaging, ICMI-BEFY, Vrije Universiteit Brussel, Laarbeeklaan 103, 1090 Brussels, Belgium; pieterjan.debie91@gmail.com (P.D.); Noemi.Berte.Declerck@vub.be (N.B.D.); Celine.Marina.Huygen@vub.be (C.M.H.); bieke.de.sloovere@hotmail.com (B.D.S.); Lukasz.Mateusiak@vub.be (L.M.); Jessica.Bridoux@vub.be (J.B.); Janik.Puttemans@vub.be (J.P.); ndevoogd@vub.be (N.D.); 2 Leiden University Medical Center, Interventional Molecular Imaging Laboratory, Department of Radiology, Leiden University, 2311 Leiden, The Netherlands; d.m.van_willigen@lumc.nl (D.v.W.); f.w.b.van_leeuwen@lumc.nl (F.W.B.v.L.)

**Keywords:** single-domain antibodies, fluorescence-guided surgery, molecular imaging, hybrid imaging

## Abstract

Intraoperative guidance using targeted fluorescent tracers can potentially provide surgeons with real-time feedback on the presence of tumor tissue in resection margins. To overcome the limited depth penetration of fluorescent light, combining fluorescence with SPECT/CT imaging and/or gamma-ray tracing has been proposed. Here, we describe the design and preclinical validation of a novel bimodal nanobody-tracer, labeled using a “multifunctional single attachment point” (MSAP) label, integrating a Cy5 fluorophore and a diethylenetriaminepentaacetic acid (DTPA) chelator into a single structure. After conjugation of the bimodal MSAP to primary amines of the anti-HER2 nanobody 2Rs15d and ^111^In-labeling of DTPA, the tracer’s characteristics were evaluated in vitro. Subsequently, its biodistribution and tumor targeting were assessed by SPECT/CT and fluorescence imaging over 24 h. Finally, the tracer’s ability to identify small, disseminated tumor lesions was investigated in mice bearing HER2-overexpressing SKOV3.IP1 peritoneal lesions. [^111^In]In-MSAP.2Rs15d retained its affinity following conjugation and remained stable for 24 h. In vivo SPECT/CT and fluorescence images showed specific uptake in HER2-overexpressing tumors with low background. High tumor-to-muscle ratios were obtained at 1h p.i. and remained 19-fold on SPECT/CT and 3-fold on fluorescence images over 24 h. In the intraperitoneally disseminated model, the tracer allowed detection of larger lesions via nuclear imaging, while fluorescence enabled accurate removal of submillimeter lesions. Bimodal nuclear/fluorescent nanobody-tracers can thus be conveniently designed by conjugation of a single-molecule MSAP-reagent carrying a fluorophore and chelator for radioactive labeling. Such tracers hold promise for clinical applications.

## 1. Introduction

Intraoperative guidance with targeted fluorescent contrast agents is an emerging tool to achieve more complete removal of malignant tissue in oncologic surgery due to its unique capability to provide real-time visual feedback to the surgeon about tumor margins and local invasion [1]. Nevertheless, the technique is limited to the detection of superficial lesions or lesions lying at most a centimeter deep in tissue, despite the deeper tissue penetration of fluorescent signals with excitation and emission in the far-red and near-infrared region as compared to a lower wavelength visible light [2]. Therefore, hybrid imaging combining fluorescence imaging with diagnostic nuclear medicine techniques has shown to be of great value in preclinical and clinical studies, as the latter can provide depth-independent information through emission of gamma-radiation [3,4,5,6,7]. The radioactive component can either be used for preoperative imaging in order to better plan surgery or can provide additional guidance during the surgery itself via gamma probe detection (real-time auditive feedback).

To design a targeted tracer that can be detected with both modalities, i.e., a bimodal tracer, the most straightforward method would be by randomly conjugating a fluorophore and chelator/prosthetic group directly to the targeting moiety. This strategy has been used for antibodies or large antibody fragments [8,9,10,11]. It does, however, lead to heterogeneous functionalization rates of the different labels, making it complex to standardize the tracer’s composition. For targeting moieties such as small antibody fragments, peptides and small molecules that only have a limited number of conjugation sites, the conjugation of multiple labels can have a more pronounced detrimental effect on their functionality and biodistribution, leading to the introduction of single bimodal labels [12]. The integration of both a fluorescent dye and a chelator for radioactive labeling into a single backbone structure has been pursued in a range of designs. One often bimodal label design is based on the so-called “multifunctional single attachment point” (MSAP) design introduced by Garanger et al., consisting of an amino acid backbone modified to bear a fluorophore, a chelator for radiolabeling, and a reactive group for conjugation [13]. Originally the MSAP label was used to create hybrid peptides such as cRGD targeting integrins and Ac-TZ14011 against CXCR4 labeled with Cy5.5/^111^In for the imaging in breast cancer models [12,14,15]. Later the backbone [16] and the fluorescent dyes in this label were varied [17,18] and it was even applied to antibodies [19,20].

**Figure 1:**
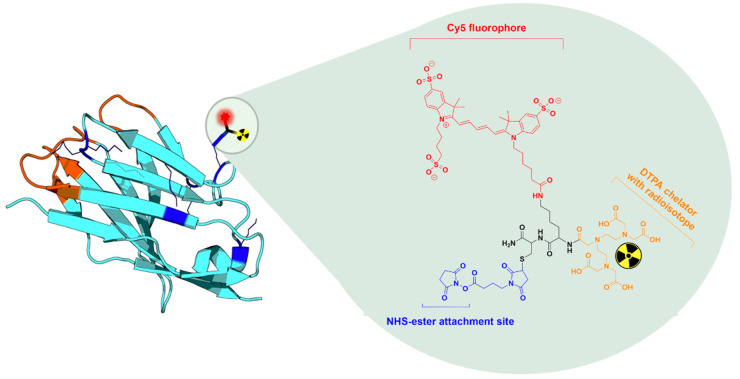
Schematic representation of the anti-HER2 Nanobody 2Rs15d being randomly labeled on its lysines via NHS-chemistry (blue) with the MSAP containing a Cy5 dye (red) and a DTPA-chelator complexed with an Indium-111 (^111^In) radioisotope (yellow). The MSAP analogue’s backbone is displayed in black.

While antibodies and peptides are commonly used tracers in molecular imaging and fluorescence-guided surgery, these compounds may have certain drawbacks. Antibodies circulate in the blood after intravenous injection for several weeks, causing high background signal and late imaging timepoints (several days) [21]. Peptides often have faster kinetics and are easier to synthetize, however, their small size makes it so their biodistribution can be heavily influenced by the chemical composition of the diagnostic labels [17]. An alternative to these targeting moieties is the use of Nanobodies. Nanobodies are the smallest stable antigen fragments, derived from camelid heavy-chain only antibodies [22]. They can be conveniently generated against molecular targets through in vivo affinity maturation and possess interesting properties for imaging [23,24,25,26]. Mainly, through their fast blood clearance and rapid, homogenous target binding, nanobody-tracers achieve high target-to-background ratio’s from 1 h post-injection in both fluorescent and nuclear molecular imaging applications [27,28,29,30,31,32]. To overcome the limitations of single labeled agents for image-guided surgery as discussed above, in this paper, we describe a bimodal nuclear/fluorescent nanobody-tracer based on a custom MSAP label (Figure 1) and report its preclinical validation for the pre- and intraoperative imaging. The human epidermal growth factor receptor 2 (HER2) was chosen as target for this proof-of-concept study given the availability of the well-characterized anti-HER2 Nanobody 2Rs15d [32].

## 2. Materials and Methods

### 2.1. Production and MSAP-Conjugation of Nanobodies

The used HER2-specific Nanobody 2Rs15d has been generated in a previous study [32] and was expressed and purified without a C–terminal hexahistidine-tag [33]. The NHS-ester functionalized MSAP molecule (Figure 1), which consists of a peptide scaffold bearing a trisulphonated Cy5 fluorophore (wavelength of maximal absorption/emission: 646/662 nm [34]) and a pentetic acid (diethylenetriaminepentaacetic acid, DTPA) chelator, was synthetized as previously described [17]. For MSAP conjugation to the primary amines on the Nanobody, 1 mg of 2Rs15d was incubated with a 1.2× molar excess of MSAP in 1 mL of 0.1 M K_2_HPO_4_ (Merck KGaA, Darmstadt, Germany) buffer at a final pH of 8.3–8.5. The conjugated Nanobody MSAP.2Rs15d was then purified using size-exclusion chromatography (SEC) on a Superdex 75 10/300GL column (GE Healthcare, Chicago, IL, USA) with 0.1 M NH_4_OAc (Acros Organics, Fair Lawn, NJ, USA) pH 7 as running buffer (0.5 mL/min). Quality control runs of the purified MSAP.2Rs15d were performed on a Superdex 75 5/150GL column with PBS pH 7.4 as running buffer (0.3 mL/min). All buffers were treated with 2 g/L chelex (Merck KGaA) before use.

One MSAP molecule contains exactly one DTPA chelator and one fluorophore, thus we can determine the MSAP degree of labeling (DOL) of the compound using the fluorophore’s concentration. Therefore, the DOL was calculated as the ratio of the fluorophore’s to the Nanobody’s concentration. This was determined by absorbance measurement at 650 and 280 nm, respectively (Nanodrop 2000 spectrophotometer, Thermo Fisher Scientific). To account for the absorbance contribution of the fluorophore and the chelator at 280 nm, the measured absorbance at 280 nm was corrected by subtracting it with 5% of the absorbance value at 650 nm.

### 2.2. Radiolabeling of MSAP-Nanobody Construct

For ^111^In-labeling, 5–120 µL of [^111^In]InCl_3_ (Curium Pharma, Petten, The Netherlands) as an aqueous solution in 0.05 M HCl (6.8–26.3 MBq) was added to the MSAP.2Rs15d construct (5 µM final concentration) and 0.2 M NH_4_OAc buffer pH 5.0, in a final volume of 35–500 µL at pH 4.5. The reaction mixture was incubated for 30 min at 50 °C [35]. After radiolabeling, the product was filtered using a 0.2 µm filter (PALL Corporation, Port Washington, NY, USA). Radiochemical purity was determined by instant thin layer chromatography (iTLC) using 0.1 M sodium citrate as mobile phase. Chemical identity of [^111^In]In-MSAP.2Rs15d (5 µg diluted in PBS with 0.1% Tween^®^ 80 detergent) was confirmed by analytical SEC on a Superdex 75 5/150GL column using PBS pH 7.4 as running buffer. Radioactivity was detected online using a Gabi detector (Elysia-Raytest, Angleur, Belgium) and was compared to the absorbance measured at 280 (Nanobody) and 650 (Cy5) nm. Stability of the bimodal tracer was furthermore evaluated by SEC after 24 h incubation in PBS at room temperature or in human serum at 37 °C.

### 2.3. In Vitro Functionality

The binding kinetics of MSAP.2Rs15d were evaluated through surface plasmon resonance (SPR) using Biacore T200 (GE Healthcare), as previously described [32]. Briefly, recombinant HER2-Fc protein was immobilized on a CM5 chip, then sensorgrams were generated for a ½ dilution series of MSAP.2Rs15d in HEPES buffered saline (HBS), ranging from 2.0 × 10^−7^ M to 1.6 × 10^−9^ M.

The specificity of the compound was further validated by a fluorescence-based cell-binding study, using HER2-overexpressing SKOV3 and HER2-negative control Chinese hamster ovarian (CHO) cells. SKOV3 and CHO cells were seeded in 24-well plates at 100,000 cells/well and incubated at 37 °C and 5% CO_2_ for 48 h. MSAP.2Rs15d was added at a final concentration of 1.1 × 10^−8^ M to wells with SKOV3 cells, CHO cells, or coincubated on SKOV3 cells with a 100-fold molar excess of unmodified 2Rs15d Nanobody. The cells were then incubated for 2 h at 4 °C, washed and remaining fluorescence in the wells was measured in the 700 nm channel of a flatbed fluorescence scanner (Odyssey, LI-COR Biosciences, Lincoln, Nebraska). For each condition three replicates were included.

To determine the affinity (K_D_) of [^111^In]In-MSAP.2Rs15d after radiolabeling, a saturation cell-binding study was performed using HER2 overexpressing SKOV3 cells. Cells were seeded in 24-well plates at 100,000 cells/well and incubated at 37 °C with 5% CO_2_ for 48 h. After ^111^In-labeling, a 1/3 dilution series (ranging from 1.0 × 10^−7^ M to 4.6 × 10^−11^ M, in triplicate) of [^111^In]In-MSAP.2Rs15d was added to the cells, either with or without a 100× molar excess of unlabeled 2Rs15d. After 1 h of incubation at 4 °C, the wells were washed, and cells were detached using 1 M NaOH. The bound activity was then measured using a gamma counter (Wizard2 2480, Perkin Elmer, Waltham, MA, USA). 

### 2.4. In Vivo Biodistribution and Tumor Targeting Potential Using Bimodal Imaging

All animal experiments were approved by the Ethical Committee for Animal Experiments of the Vrije Universiteit Brussel (project nr. 15-272-5). The mice were housed in individually ventilated cages at 19–24 °C in 40–60% humidity with a light/dark cycle of 14/10 h. Low fluorescence food pellets (Teklad 2016, Envigo, Indianapolis, IN, USA) and water were provided ad libitum.

#### 2.4.1. Longitudinal Biodistribution Study in Subcutaneous Xenografts

SKOV3 (10 × 10^6^ cells) or HER2-negative MDA-MB-435S (2 × 10^6^ cells) subcutaneous tumors were implanted on the right flank of female athymic nude Crl:NU-Foxn1^nu^ mice (Charles River, *n* = 3 per group), and grown until the tumors for all animals within a group reached a volume between 100 and 500 mm³.

Of [^111^In]In-MSAP.2Rs15d 7.5 µg (12.3 ± 0.5 MBq, corresponding to 1 nmol MSAP, and apparent molar specific activity of 13.5 ± 0.6 GBq/µmol) was injected via the tail vein of either SKOV3 or MDA-MB-435S xenograft bearing mice. Consecutive single photon emission computed tomography/computed tomography (SPECT/CT) scans were performed at 1, 4, and 24 h post-injection, and after each scan, the same animal was subjected to fluorescence imaging. After the final timepoint, animals were killed by cervical dislocation for further ex vivo biodistribution studies. Fluorescence imaging of individual organs and tissues was performed, whereafter the organs and tissues were weighed, and their radioactive signal measured using a gamma counter (Wizard2 2480, Perkin Elmer). Results were decay-corrected and expressed as percentage of injected dose per cm^3^ (%ID/cm^3^).

#### 2.4.2. Image-Guided Resection of Intraperitoneally Disseminated Tumor Lesions

Luciferase-expressing SKOV3.IP1 cells (0.5 × 10^6^), kindly provided by Prof. Marc Bracke (UGent, Belgium), were intraperitoneally injected in Crl: NU-Foxn1^Nu^ mice (Charles River; *n* = 3) [30,36]. Tumor growth was followed-up using bioluminescence imaging for 30 days. [^111^In]In-MSAP.2Rs15d (10.5 ± 0.5 MBq, 7.5 µg corresponding to 1 nmol MSAP, and apparent molar specific activity of 11.6 ± 0.5 GBq/µmol, intravenously) and luciferin (150 mg/kg, intraperitoneally) were administered 1 h and 10 min, respectively prior to SPECT/CT imaging. Then, the animals were killed via cervical dislocation and peritoneal tumor lesions were resected under fluorescence guidance. Finally, fluorescence and radioactive signals of all confirmed tumor lesions, and of the major peritoneal organs were measured ex vivo as described in 2.4.1 and 2.5. Bioluminescence imaging (BLI) (PhotonIMAGER^TM^ Optima, Biospace, Nesles la Vallée, France) was used to confirm the tumorous character of resected lesions.

### 2.5. Imaging Protocols

During all imaging procedures, for intravenous injections and for cervical dislocation, mice were anaesthetized with isoflurane gas (5% for induction, 2% for maintenance during the scan, 0.5–1.0 mL/min oxygen flow rate). MicroSPECT/CT imaging was performed using a Vector^+^ system (Milabs) equipped with a general-purpose rat/mouse 1.5 mm 75 pinhole collimator. Scans were performed in spiral mode with 6 bed positions and an acquisition time of 200 s per bed position. For image reconstruction, 2 subsets and 4 iterations were used, with a voxel size of 0.4 mm in U-SPECT-Rec software (Milabs). The CT scan was made in 1 bed position, with a duration of 146 s at 60 kV and a pixel size of 80 µm. Further quantitative image analysis was performed with AMIDE software (calculation of percent injected dose per cm^3^ (%ID/cm^3^) in region-of-interests (ROIs)), and 3D images were prepared in Osirix software (Pixmeo, Bernex, Switzerland). Radioactive tumor-to-muscle (TMR^rad^) ratios were determined by dividing the tumor’s %ID/cm^3^ by the muscle’s %ID/cm^3^.

Fluorescence imaging was performed using a KIS700 camera (Kaer Labs, Nantes, France), an open surgical system with resolution of 1920 × 1200, excitation wavelength of 640 nm and emission light collection above 665 nm (high pass). Background fluorescence (measured without excitation) was subtracted from the images and analysis was performed using ImageJ. For the different ROIs, mean fluorescent intensity (MFI) was calculated. Fluorescent TMR (TMR^fluo^) ratios were determined by dividing the tumor’s MFI values by the muscle’s MFI values.

### 2.6. Statistical Analysis

For the fluorescence in vitro functionality assay, results were compared using an ordinary one-way ANOVA, corrected for multiple comparisons. Uptake between HER2-expressing and HER2-negative tumors was compared using an unpaired Student’s *t*-test. The correlation between nuclear and fluorescent signals was investigated by linear regression. Statistical analyses were performed using Prism 7 (Graphpad Software, San Diego, CA, USA). All data on the graphs is displayed as the mean ± SD.

## 3. Results

### 3.1. Production, MSAP Conjugation, and Radiolabeling of Nanobodies

Following MSAP conjugation to the 2Rs15d Nanobody, SEC analysis confirmed the purity of MSAP.2Rs15d. The compound eluted as a single peak with a retention time of 6–7 min (as expected for a Nanobody), and absorbing at both 280 and 650 nm. The average DOL was determined to be 1.1 MSAP molecules per Nanobody.

**Figure 2:**
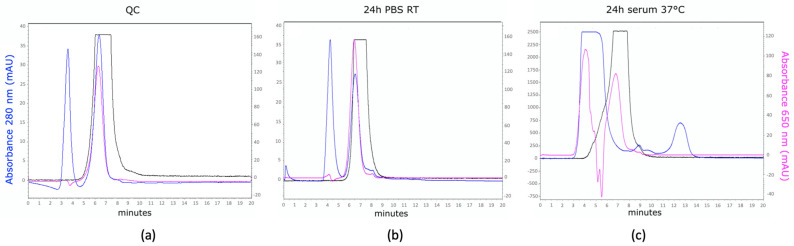
Quality control and stability size-exclusion chromatography (SEC) profiles of [^111^In]In-MSAP.2Rs15d. (**a**) SEC profile of MSAP.2Rs15d after radiolabeling with ^111^In. (**b**) SEC profile of [^111^In]In-MSAP.2Rs15d after 24 h incubation in PBS. (c) SEC profile of [^111^In]In-MSAP.2Rs15d after 24 h incubation in human serum at 37 °C. The blue line denotes absorption at 280 nm, the pink line denotes absorption at 650 nm, and the black line represents the radioactive signal intensity. Both in panel (**a**,**b**), [^111^In]In-MSAP.2Rs15d is dissolved in PBS 0.05% Tween^®^ 80; the additional peak seen around 4 min is attributed to the absorbance of Tween 80 at 280 nm. In panel (**c**), the absorbance of the Nanobody at 280 nm is negligible compared to the intense signal at 280 nm of the serum proteins. Therefore, the stability of the compound is determined based on the presence of the radioactive signal peak and the MSAP absorption peak at 650 nm at 6–7 min, comparable to the profiles in panel (**a**,**b**). Furthermore, serum proteins with a retention time of 3-5 min exhibit some autofluorescence at 650 nm, explaining the peak in 650 nm absorbance at 3–5 min.

MSAP.2Rs15d could be successfully ^111^In-labeled, yielding a radiochemical purity of >95% as determined by iTLC. Further quality controls (QC) performed by radio-SEC, confirmed that the radioactive signal matched the retention times of MSAP.2Rs15d (absorbance at 280 and 650 nm) (Figure 2a), and that the tracer remained stable up to at least 24 h incubation in PBS or serum as no degradation of the protein, fluorescent, and/or radioactive signal was seen (Figure 2b,c)

### 3.2. In Vitro Functionality

**Figure 3:**
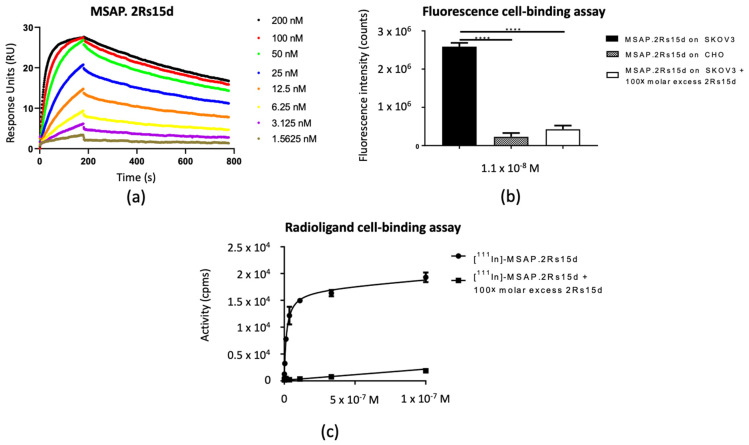
In vitro functionality assays. (**a**) SPR graphs of MSAP.2Rs15d on the immobilized HER2-Fc protein. (**b**) Fluorescence cell-binding assay of MSAP.2Rs15d at a fixed concentration of 1.1 × 10^-8^ M on HER2-expressing SKOV3 cells or HER2-negative CHO cells (**** *p* < 0.001). Data are represented as mean ± SD (*n* = 3 replicates per condition). (**c**) Saturation cell-binding assay (radioactive) demonstrating the specificity and high affinity of the [^111^In]In-MSAP.2Rs15d for the HER2-expressing SKOV3 cells. Data are represented as mean ± SD (*n* = 3 replicates per condition).

SPR measurements revealed the dissociation constant (K_D_) of the MSAP.2Rs15d construct to be 5.0 ± 0.1 × 10^−9^ M (Figure 3a). Specificity of the construct was further demonstrated by the significantly higher binding to HER2-expressing SKOV3 cells than to non-HER2-expressing CHO cells, or SKOV3 cells incubated with an excess of unlabeled 2Rs15d (Figure 3b). With a K_D_ of 1.6 ± 0.2 × 10^−9^ M as determined via a saturation binding assay on SKOV3 cells, it was confirmed that also after ^111^In-labeling, the affinity of the Nanobody was not negatively affected (Figure 3c). The small difference in K_D_ measured using SPR and cell-binding studies can be explained by the use of purified recombinant HER2 protein in the case of SPR, and high HER2-overexpressing cells for the radioligand-binding assay. Nevertheless, both values are in the low nanomolar range and comparable to that of the unconjugated 2Rs15d (3.2 ± 0.1 × 10^−9^ M) (Figure S1).

### 3.3. In Vivo Biodistribution and Tumor Targeting Potential Using Bimodal Imaging

#### 3.3.1. Longitudinal Biodistribution in Subcutaneous Xenografts

**Figure 4:**
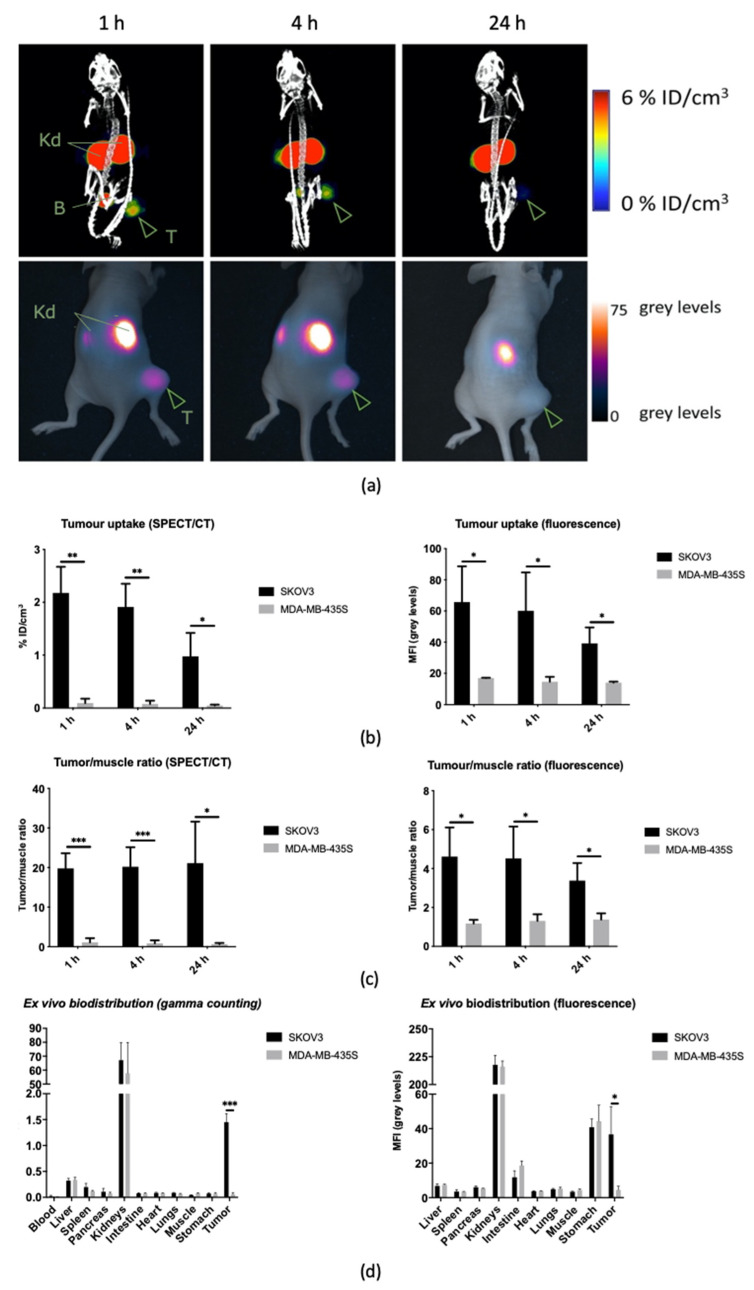
(**a**) Representative SPECT/CT (top) and fluorescence (bottom) images of a SKOV3 bearing mouse at 1 h, 4 h, and 24 h post-injection of [^111^In]In-MSAP.2Rs15d. Specific uptake can be observed in the tumor (T), with little to no non-specific uptake, except for in the kidneys (Kd) and the bladder (B). This results in the specific and high-contrast imaging of HER2-positive subcutaneous tumors. (**b**) In vivo quantification of [^111^In]In-MSAP.2Rs15d tumor uptake, based on SPECT/CT (left) or fluorescence (right). SPECT/CT data is represented as % of the initial injected dose per cm^3^ of tissue (%ID/cm^3^), whereas the fluorescence data are represented as the mean fluorescence intensity (MFI) of the tumor area (in grey levels) (** *p* < 0.01, * *p* < 0.05). All data is represented as mean ± SD (*n* = 3 mice per group, *N* = 6). (**c**) In vivo tumor-to-muscle ratios over time measured by SPECT/CT (left) or fluorescence (right) (*** *p* < 0.005, * *p* < 0.05). All data is represented as mean ± SD (*n* = 3 mice per group, *N* = 6). (**d**) Ex vivo biodistribution of [^111^In]In-MSAP.2Rs15d in SKOV3 and MDA-MB-435S xenograft bearing mice at 24 h post injection. Determined by gamma counting (left) as % injected dose per gram (%ID/g) or by fluorescence measurement (right) as MFI. Both biodistribution profiles match, except for the elevated signal in gastrointestinal organs measured by fluorescence, due to the mouse chow (*** *p* < 0.005, * *p* < 0.05). All data is represented as mean ± SD (*n* = 3 mice per group, *N* = 6).

As shown in Figure 4, SPECT/CT and fluorescence images coincided, and revealed an analogous biodistribution pattern for [^111^In]In-MSAP.2Rs15d at 1 h, 4 h, and 24 h post-injection, with low non-specific uptake in untargeted organs, except for the kidneys due to renal retention of the tracer. SKOV3 tumors could be clearly distinguished as soon as 1 h post-injection, while no uptake in control MDA-MB-435S xenografts was seen (Figure 4a and Figure S2). This was confirmed by quantification of the tumor uptake and tumor-to-muscle ratios (TMRs) based on the SPECT/CT images, yielding significant differences between SKOV3 and MDA-MB-435S at all timepoints (Figure 4b,c). More specifically, in SKOV3 xenografted mice a TMR^rad^ of 19.8 ± 3.8 was achieved within 1 h and preserved over 24 h (21.1 ± 10.5). The absolute specific uptake did gradually decrease over time (Figure 4b) from 2.2 ± 0.5 %ID/cm^3^ at 1 h to 1.0 ± 0.4 %ID/cm^3^ at 24 h post-injection. The TMR^rad^ for MDA-MB-435S control tumors was only 1.1 ± 1.0 (*p* < 0.05). On the fluorescent images, tumor uptake in SKOV3 xenografted mice could be clearly visualized with TMR^fluo^ of 4.6 ± 1.5 and 4.5 ± 1.6 at respectively 1 and 4 h. These values were lower than for SPECT/CT imaging in consequence of signal attenuation by tissue and background autofluorescence.

The in vivo findings were further confirmed by the ex vivo biodistribution analysis after 24 h (Figure 4d, Tables S1 and S2). Indeed, the semiquantitative fluorescent data reflects the trend seen in the quantitative data obtained through gamma counting, with exception of the elevated fluorescent signals in the intestines and stomach (due to mouse chow [37]). The overall tumor uptake after 24 h in SKOV3 xenografts was measured to be 1.5 ± 0.2 %ID/g, which is similar to the in vivo measured data. Table S1 further illustrates the high tumor-to-organ ratios.

#### 3.3.2. Image-Guided Resection of Intraperitoneally Disseminated Tumor Lesions

**Figure 5:**
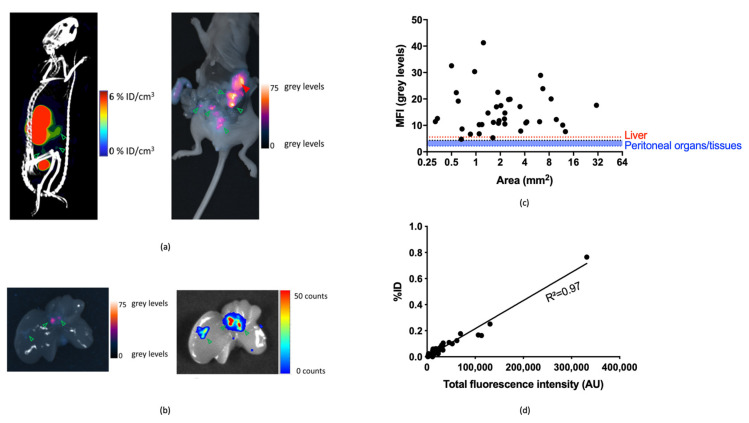
(**a**) Representative SPECT/CT (left) and fluorescence (right) scan of a mouse bearing intraperitoneally disseminated SKOV3.IP1 tumor lesions at 1 h post-injection of [^111^In]In-MSAP.2Rs15d. Tumor tissue is denoted by green arrows, the stomach is indicated by a red arrow. (**b**) Ex vivo liver with tumor nodules visualized by fluorescence imaging (left) and confirmed by BLI (right). Some non-specific fluorescence is seen due to the gallbladder. Tumor tissue is denoted by green arrows. (**c**) Mean fluorescence intensity (MFI) of individual SKOV3.IP1 tumor lesions as a function of their size. MFI is displayed relative to the average MFI of relevant peritoneal organs and tissues (spleen, pancreas, intestine, and muscle; blue line) and of the liver (red line) (*n* = 3 mice per group, *N* = 3). (**d**) Correlation of radioactive and fluorescent signals. The total %ID of radioactivity is plotted against the fluorescence intensity over the whole tumor area. A linear relationship is seen between both modalities, indicating a good correspondence of the signals (*n* = 3 mice per group, *N* = 3).

SPECT/CT scans (1 h post-injection) could be used to visualize the largest tumor masses (Figure 5a), however, smaller tumor nodules could not be visualized because of the inherent limited resolution and sensitivity of the imaging technique and shine through of the nearby kidneys. Subsequent opening of the abdominal cavity allowed in situ fluorescence-based visualization and fluorescence-guided removal of even submillimeter tumor lesions that were spread at the surface of the animal’s peritoneum (38 lesions were removed in total, Figure 5a–c). Ex vivo analysis of BLI-confirmed tumor lesions was then used to correlate the uptake of fluorescence and radioactive signals in the resected lesions. The %ID of radioactivity in resected tumor lesions was plotted against their total fluorescence signal (Figure 5d), and after fitting a line with linear regression, an R^2^ value of 0.97 was obtained, which indicates the good correlation between both modalities. The biodistribution and tumor-to-organ ratio’s obtained through ex vivo fluorescence imaging and gamma counting is displayed in Figure S3 and Table S3.

## 4. Discussion

Fluorescence surgical guidance has potential to improve the detection and removal of cancerous tissue, however, the technique is held back by unfavorable interaction of light and tissue (attenuation and scattering). Therefore, the use of bimodal labels such as the MSAP analogues, combining fluorescence and nuclear imaging may help overcome this limitation. Such a combination has been shown to allow pre/intraoperative detection of disease in a depth-independent manner and thus facilitates surgical navigation [3]. We here demonstrated that this concept can also be used to advance the use of Nanobodies in molecular imaging towards such bimodal applications.

We showed that an MSAP analogue carrying both a Cy5 fluorescent dye and a DTPA chelator for ^111^In-labeling could be readily conjugated to a HER2-specific Nanobody without impacting its affinity or its pharmacokinetics. Administration of a single dose of the tracer resulted in high-contrast and specific visualization of HER2-expressing tumors via both SPECT/CT and fluorescence imaging (1–24 h p.i.), and with the exception of the kidneys, little to no non-specific uptake in HER2-negative tumors or non-targeted tissues. This corroborates with previous biodistribution profiles obtained after labeling of the same Nanobody with various PET, SPECT, or therapeutic radioisotopes [32,33,38,39,40,41], and is in stark contrast with the drastic effect random labeling with the heptamethine fluorophore IRDye800CW has on the biodistribution of Nanobodies (high non-specific uptake and hepatic clearance [29]). The high fluorescent signal in the kidneys as observed in mice will most likely pose no problems in patients due to the attenuation of the signal by perinephric fat. However, the radioactive signal originating from the kidneys may create a significant background signal and affect adjacent tumor lesion detection. The use of Gelofusin or positively charged amino acids have been shown to reduce kidney retention in previous studies and could possibly be applied to counteract this limitation [42]. In the future, site-specific conjugation methods using cysteine-maleimide chemistry [41] or using the enzyme Sortase [40] could be considered to further standardize the tracer’s composition if needed. Additional improvements can be found in minimizing the bimodal labels and using chelates that allow radiolabeling with ^99m^Tc, a radioisotope with more translational potential [43].

The proof-of-concept study in a murine model of intraperitoneally disseminated cancer showed that addition of a nuclear modality to the fluorescent component can further extend the potential of Nanobody-based imaging. Besides improvement of the characterization process and enabling a more exact determination of the tracer’s biodistribution, [^111^In]In-MSAP.2Rs15d could be used for both preoperative planning, and for precise and sensitive guidance during the actual surgical intervention. Indeed, preoperative nuclear medicine imaging can provide useful information on the anatomical localization of the tumor lesions and lymph node metastases [44]. Radioguided surgery solutions in the form of intraoperative gamma tracing can help to further guide the surgeon towards cancerous lesions, a concept that is currently being explored for prostate cancer [45]. A limitation of the study is certainly the choice of HER2 as biomarker, given its overexpression in a restricted number of cancer types and its intratumoral heterogeneity [46].

## 5. Conclusions

In this study we described the preparation of a bimodal nuclear/fluorescent Nanobody-tracer through the convenient conjugation of a single-molecule MSAP-reagent carrying both a fluorophore and chelator for radioactive labeling. The Nanobody-tracer possessed an adequate biodistribution profile enabling fast and high-contrast nuclear and fluorescent imaging with low background. Such a tracer holds promise for clinical application in the context of image-guided surgery as was demonstrated by a proof-of-concept study in which intraperitoneally tumor lesions could be localized preoperatively using SPECT/CT, and then precisely excised via intraoperative fluorescence imaging.

## Data Availability

Data sharing is not applicable for this article. All data is contained within the article or supplementary material. The data presented in this study are available in ‘The Design and Preclinical Evaluation of a Single-Label Bi-modal Nanobody Tracer for Image-Guided Surgery—Supplementary data’.

## Supplementary Materials

The following are available online at https://www.mdpi.com/2218-273X/11/3/360/s1, Figure S1: SPR graphs of reference unconjugated 2Rs15d on immobilized HER2-Fc protein, Figure S2: SPECT/CT (*a*) and fluorescence (*b*) images of mice bearing MDA-MB-435S xenografts, Table S1: Tumor-to-organ ratios calculated from ex vivo biodistribution at 24 h post-injection of [^111^In]-MSAP.2Rs15d in mice bearing subcutaneous SKOV3 xenografts, Table S2: Tumor-to-organ ratios calculated from ex vivo biodistribution at 24 h post-injection of [^111^In]-MSAP.2Rs15d in mice bearing subcutaneous MDA-MB-435S xenografts, Figure S3: Ex vivo biodistribution of mice bearing SKOV3.IP1 intraperitoneally disseminated tumor lesions as determined by SPECT/CT and fluorescence imaging, Table S3: Tumor-to-organ ratios calculated from ex vivo biodistribution at 1 h post-injection of [^111^In]-MSAP.2Rs15d in mice bearing intraperitoneally disseminated SKOV3.IP1 tumors, Figure S4: Ex vivo imaging of resected pancreas from SKOV3.IP1 intraperitoneally disseminated tumor bearing mouse.
