# Supplementary material for: The Design and Preclinical Evaluation of a Single-Label Bimodal Nanobody Tracer for Image-Guided Surgery
Source: Biomolecules. 2021 Feb 26;11(3):360. doi: 10.3390/biom11030360 (PMC7996797; doi:10.3390/biom11030360)
Supplement: Supplementary file 1 [file biomolecules-11-00360-s001.pdf]

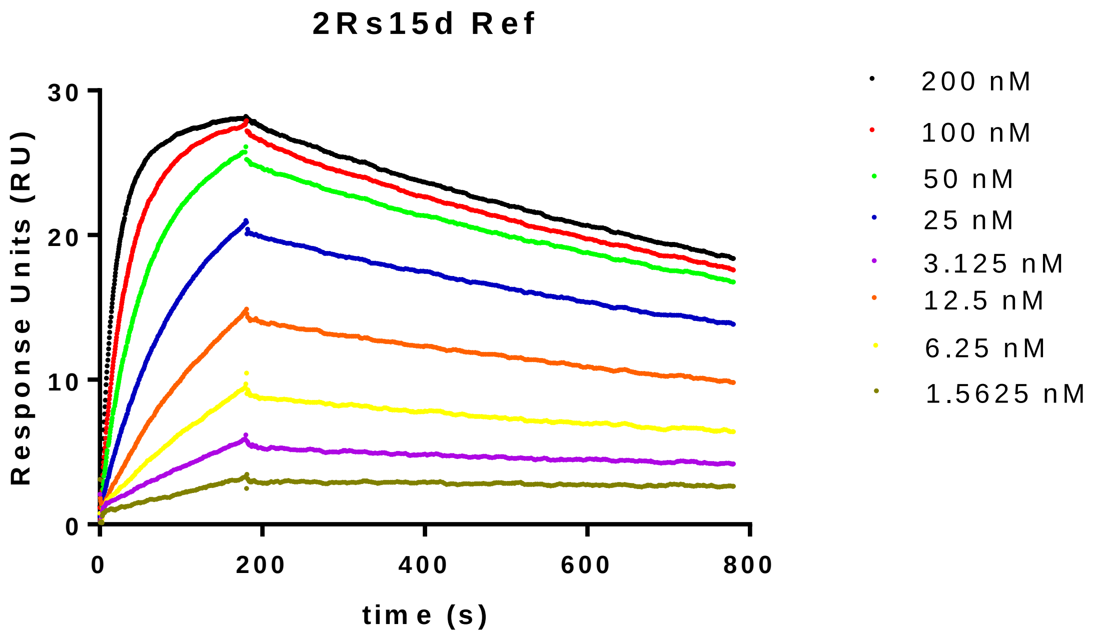

Figure S1: SPR graphs of reference unconjugated 2Rs15d on immobilized HER2-Fc protein. Affinity determined as 3.2 ± 0.1 nM.

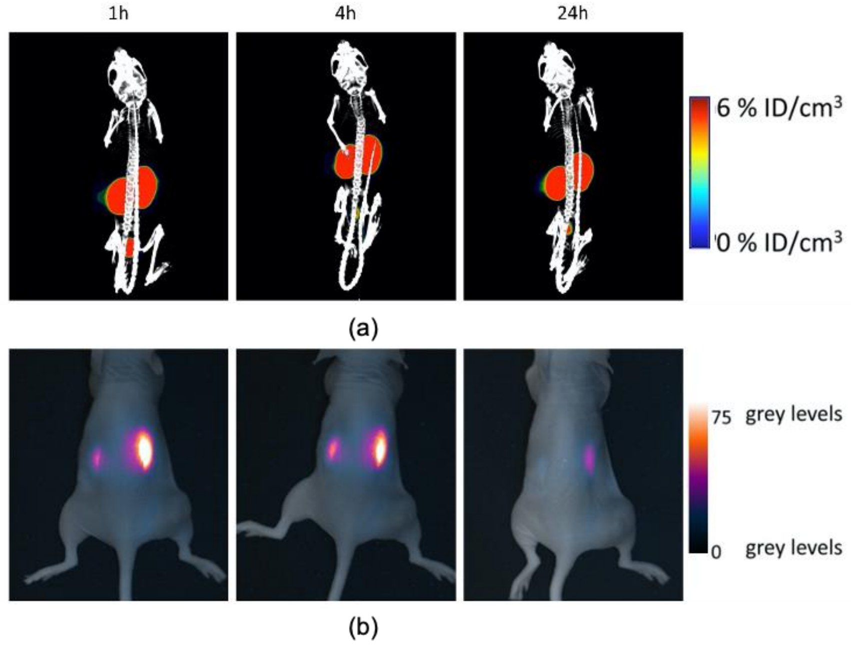

Figure S2: SPECT/CT (a) and fluorescence (b) images of mice bearing MDA-MB-435S xenografts at different timepoints post-injection of [^{111}In]-MSAP.2Rs15d.

Table S1: Tumour-to-organ ratios calculated from *ex vivo* biodistribution at 24h post-injection of [^{111}In]-MSAP.2Rs15d in mice bearing subcutaneous SKOV3 xenografts (N = 3).

Tumour-to-organ ratio	Gamma-counting	Fluorescence imaging
Blood	54.5 ±6.2	NA
Liver	4.5 ± 0.5	5.4 ± 2.5
Spleen	7.4 ± 0.8	10.3 ± 5.5
Pancreas	13.3 ± 1.5	6.0 ± 2.7
Kidney	0.020 ± 0.002	0.1 ± 0.1
Intestine	18.7 ± 2.1	3.1 ± 1.7
Heart	16.8 ± 1.9	9.8 ± 4.4
Lungs	16.8 ± 1.9	7.5 ± 3.4
Muscle	32.3 ± 3.6	10.8 ± 4.8
Stomach	19.6 ± 2.2	0.9 ± 0.4
Tumor	1 ± 0.1	1.0 ± 0.6

Table S2: Tumour-to-organ ratios calculated from *ex vivo* biodistribution at 24h post-injection of [^{111}In]-MSAP.2Rs15d in mice bearing subcutaneous MDA-MB-435S xenografts (N = 3).

Tumour-to-organ ratio	Gamma-counting	Fluorescence imaging
Blood	5.9 ± 3.5	NA
Liver	0.2 ± 0.1	0.6 ± 0.3
Spleen	0.5 ± 0.3	1.3 ± 0.6
Pancreas	0.7 ± 0.4	0.8 ± 0.4
Kidney	0.0009 ± 0.0005	0.015 ± 0.008
Intestine	0.8 ± 0.5	0.2 ± 0.1
Heart	0.7 ± 0.4	1.2 ± 0.6
Lungs	0.8 ± 0.5	0.8 ± 0.4
Muscle	0.7 ± 0.4	1.0 ± 0.5
Stomach	0.8 ± 0.5	0.10 ± 0.05
Tumor	1.00 ± 0.09	1.0 ± 0.7

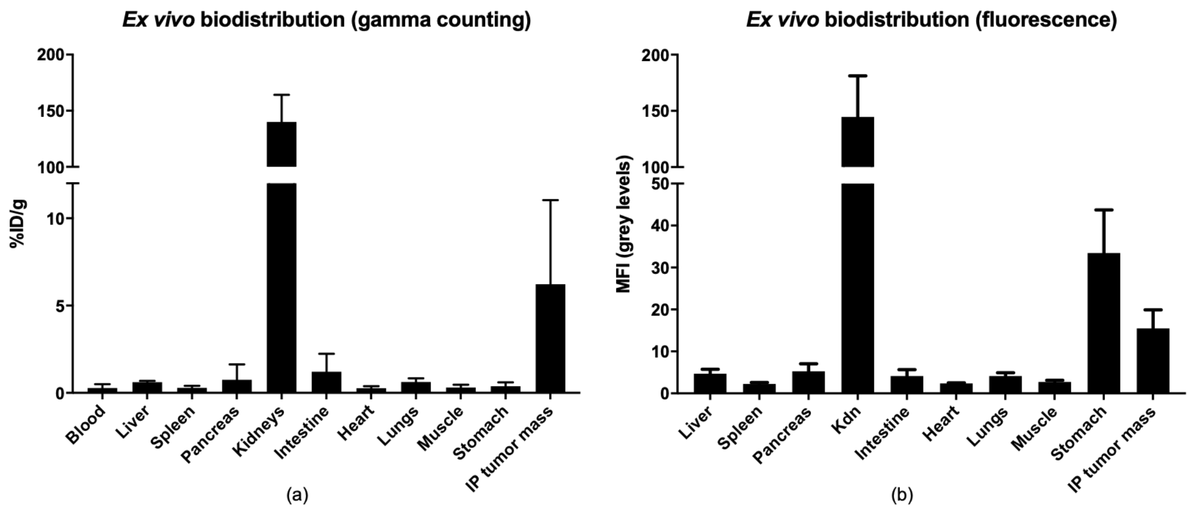

Figure S3: *Ex vivo* biodistribution of mice bearing SKOV3.IP1 intraperitoneally disseminated tumour lesions as determined by SPECT/CT (a) and (b) fluorescence imaging. All data is represented as mean ± SD (N = 3). Limited uptake in pancreas and intestine can be attributed to the presence of tumour small lesions in these organs.

Table S3: Tumour-to-organ ratios calculated from *ex vivo* biodistribution at 1h post-injection of [^{111}In]-MSAP.2Rs15d in mice bearing intraperitoneally disseminated SKOV3.IP1 tumours (N = 3).

Tumour-to-organ ratio	Gamma counting	Fluorescence imaging
Blood	65.8 ± 47.5	NA
Liver	10.5 ± 7.0	3.4 ± 1.0
Spleen	22.1 ± 10.8	7.3 ± 2.4
Pancreas	20.6 ± 13.4	3.1 ± 0.9
Kidney	0.1 ± 0.1	0.1 ± 0.1
Intestine	6.1 ± 2.5	3.9 ± 0.8
Heart	23.1 ± 9.2	6.5 ± 1.6
Lungs	10.0 ± 5.2	3.9 ± 1.1
Muscle	18.9 ± 3.8	6.0 ± 2.1
Stomach	18.7 ± 11.4	0.5 ± 0.1

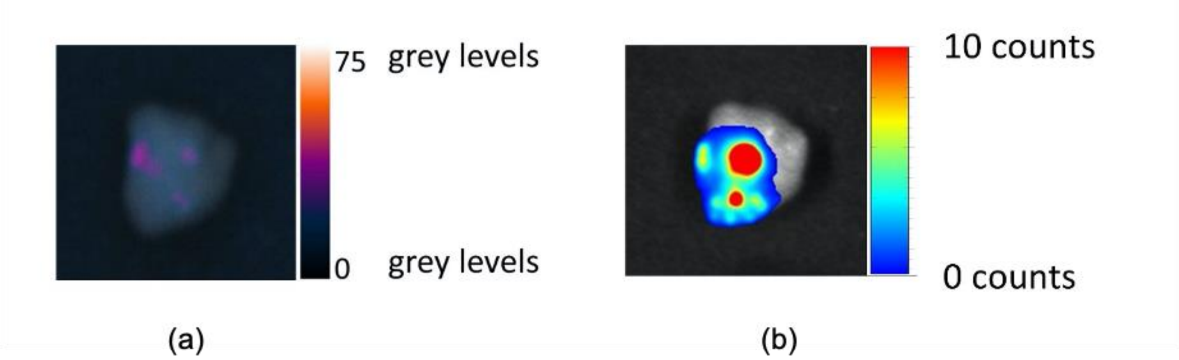

Figure S4: *Ex vivo* imaging of resected pancreas from SKOV3.IP1 intraperitoneally disseminated tumour bearing mouse. Presence of tumour nodules identified by fluorescence imaging (a) and confirmed by bioluminescence imaging (b).
